# Structural effects on the luminescence properties of CsPbI_3_ nanocrystals†

**DOI:** 10.1039/d2nr06345j

**Published:** 2023-02-14

**Authors:** Olivera Vukovic, Giulia Folpini, E Laine Wong, Luca Leoncino, Giancarlo Terraneo, Munirah D. Albaqami, Annamaria Petrozza, Daniele Cortecchia

**Affiliations:** a Centre for Nano Science and Technology (CNST@PoliMi), Istituto Italiano di Tecnologia Via Pascoli 70 Milan 20133 Italy annamaria.petrozza@iit.it daniele.cortecchia2@unibo.it; b Molecular Materials and Nanosystems & Institute for Complex Molecular Systems, Eindhoven University of Technology 5600 MB Eindhoven The Netherlands; c Université de Pau & Pays Adour, CNRS, IPREM UMR 5254 2 Avenue du Président Angot Pau F-64053 France; d Electron Microscopy Facility, Istituto Italiano di Tecnologia Via Morego 30 Genova 16163 Italy; e Laboratory of Supramolecular and Bio-Nanomaterials (SupraBioNanoLab), Department of Chemistry, Materials, and Chemical Engineering “Giulio Natta”, Politecnico di Milano via L. Mancinelli 7 20131 Milano Italy; f Chemistry Department, College of Science, King Saud University Riyadh 11451 Saudi Arabia

## Abstract

Metal halide perovskite nanocrystals (NCs) are promising for photovoltaic and light-emitting applications. Due to the softness of their crystal lattice, structural modifications have a critical impact on their optoelectronic properties. Here we investigate the size-dependent optoelectronic properties of CsPbI_3_ NCs ranging from 7 to 17 nm, employing temperature and pressure as thermodynamic variables to modulate the energetics of the system and selectively tune the interatomic distances. By temperature-dependent photoluminescence spectroscopy, we have found that luminescence quenching channels exhibit increased non-radiative losses and weaker exciton–phonon coupling in bigger particles, in turn affecting the luminescence efficiency. Through pressure-dependent measurements up to 2.5 GPa, supported by XRD characterization, we revealed a NC-size dependent solid–solid phase transition from the γ-phase to the δ-phase. Importantly, the optical response to these structural changes strongly depends on the size of the NC. Our findings provide an interesting guideline to correlate the size and structural and optoelectronic properties of CsPbI_3_ NCs, important for engineering the functionalities of this class of soft semiconductors.

## Abstract 2

By studying the temperature and pressure response of CsPbI_3_ nanocrystals, we demonstrated that the lattice deformation mechanism strongly affects the material's bandgap and recombination dynamics in nanocrystals with different sizes.

## Graphical abstract

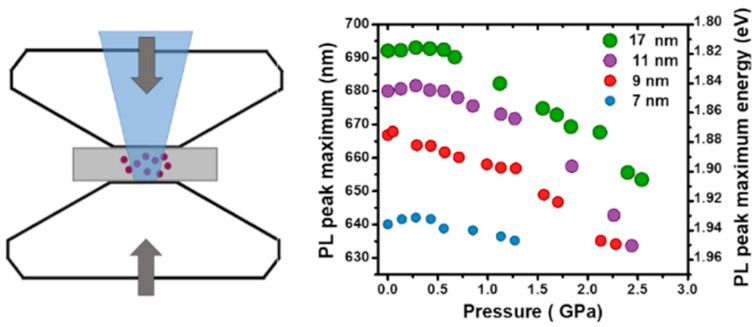

## Introduction

The facile and scalable synthesis of colloidal perovskite nanocrystals (NCs), combined with the tunability of their optoelectronic properties, high photoluminescence quantum yield (PLQY), and defect tolerance,^1^ makes them promising candidates for next-generation photonic devices from photovoltaic cells to light-emitting diodes.^2–6^ Spectral tunability through the whole visible range can be achieved through chemical substitution and NC size engineering. NCs’ size plays a critical role not only by affecting the optical and electronic characteristics through quantum confinement, but also by changing the surface-to-volume ratio and consequently the structural properties,^7^ given that the relaxed coordination geometry at the surface can locally induce different structural arrangements.^8^ The relatively soft nature of the metal halide lattice can result in a wide range of structural distortions and defects that also have a huge impact on the optoelectronic properties.^9–12^ This effect is particularly severe in CsPbI_3_ NCs, where the tensile strain induced by the high surface-to volume-ratio allows the stabilization of their perovskite structure at room temperature, which would be otherwise thermodynamically unstable towards the conversion to the non-perovskite yellow δ-phase under bulk conditions.^13,14^ Therefore, an accurate size-dependent study of perovskite NCs is required to single out the complex contribution of the particle size, quantum confinement, structural distortions, defect losses, and phonon scattering, retrieving fundamental structure–property relationships that allow a more precise and educated material's design. Despite the advances in understanding the photophysics of perovskite NCs,^7,15–25^ works reporting the size-dependent investigation of their optical and structural properties are still very limited;^7,13^ this is likely due to the challenges posed by the standard hot injection synthesis, where scarce control over the NC nucleation and growth makes it difficult to achieve narrow size distributions over a wide range.

In this work, we employed the hot injection synthetic method and adapted a size-selective precipitation strategy^13^ to controllably isolate CsPbI_3_ NCs with sizes ranging between the strong and weak quantum confinement regime (7–17 nm) and combined photoluminescence (PL) spectroscopy and X-ray diffraction (XRD) characterization to elucidate their size-dependent structural and photophysical properties. Low-temperature luminescence measurements allowed us to investigate the PL quenching channels in NCs of different sizes, identifying the critical role of exciton–phonon coupling and surface defect passivation by the organic ligands. By loading the NCs in a diamond anvil cell (DAC), we then performed pressure-dependent characterization up to 2.5 GPa, which allowed us to modulate the interatomic distances and study the effect of structural distortion and quantum confinement independently of size-related effects; we attribute the pressure response (PL peak shift, change in recombination dynamics and quenching at high pressures) to the lattice deformation mechanism, finding a different structural evolution pattern for the smallest size NCs. By highlighting the key role of particles’ size, our findings shed light on the fundamental relationship between the structural and optoelectronic properties of CsPbI_3_ NCs that can likely be applied to a wide range of perovskite systems.

## Results and discussion

**Fig. 1:**
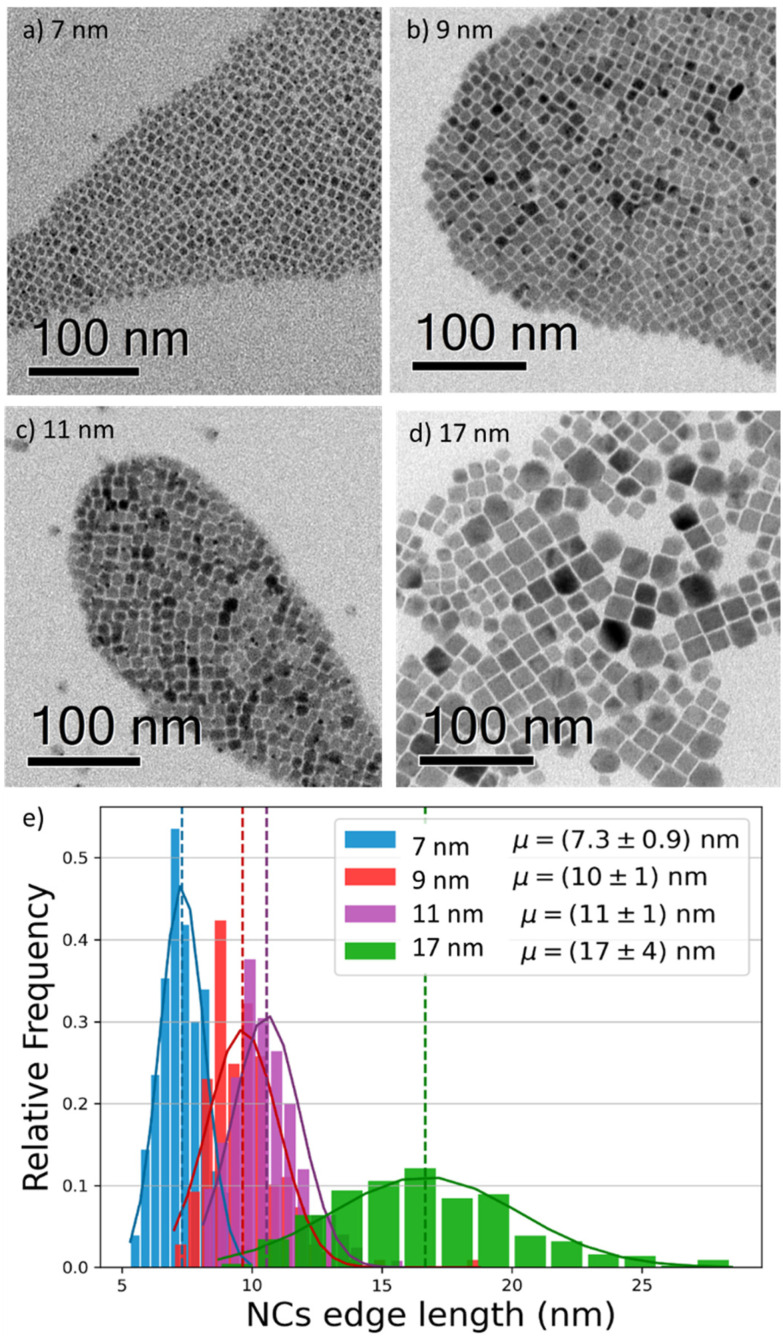
(a–d) Bright field transmission electron microscopy (TEM) images of four different CsPbI_3_ NCs, revealing their cubic shape and sizes of 7, 9, 11, and 17 nm. The solutions have low polydispersion and narrow size distribution. (e) Size distribution histogram of the imaged NCs (statistics: 200 per size), showing narrow distributions (±1 nm) for the smaller sizes, and a broader distribution (±4 nm) for the 17 nm NCs.

To investigate the effect of the particle size on the luminescence properties of NCs, we aimed at synthesizing NCs in a broad range of sizes between 7 and 17 nm; given that the Bohr radius for CsPbI_3_ is about 12 nm,^26^ this range allows us to span from a weak to strong quantum confinement regime. To meaningfully study different sizes, the size selectivity provided by the hot injection method needs to be better than the typical size dispersion in the solution (>10%).^1,13^ Since the reaction kinetics is very fast, usually completed within 5 seconds, the control of nucleation and growth is the main challenge. Using a size-selective precipitation purification method,^27^ we were able to successfully achieve low polydispersion and narrow size distributions (Fig. 1e): for the smaller particles (7 to 11 nm) the dispersion has a variance of 1 nm, while for the larger particles, the distribution is centered on 17 nm and has a dispersion of 4 nm. The representative transmission electron microscopy (TEM) images shown in Fig. 1 confirmed the formation of CsPbI_3_ NCs with a cubic shape and size of 7, 9, 11, and 17 nm. Bulk CsPbI_3_ has three known black phases (α, β, and γ), with a slightly different symmetry, which form at high temperatures of up to 330 °C. Due to the small size of Cs^+^, such perovskite phases made of corner-sharing octahedra are not stable at room temperature, and quickly convert into a non-perovskite orthorhombic structure, known as the δ or yellow phase. The structural strain arising from the high surface-to-volume ratio in NCs allows the stabilization of the perovskite black phase under ambient conditions also, but the identification of the exact symmetry has been a matter of debate.^18,28^ To confirm the structure of our nanoparticles, we performed X-Ray diffraction (XRD) on drop-casted samples (Fig. S1a†), and it indicated the formation of the orthorhombic γ-phase (Fig. S1b†), in agreement with other recent results.^17,29^

**Fig. 2:**
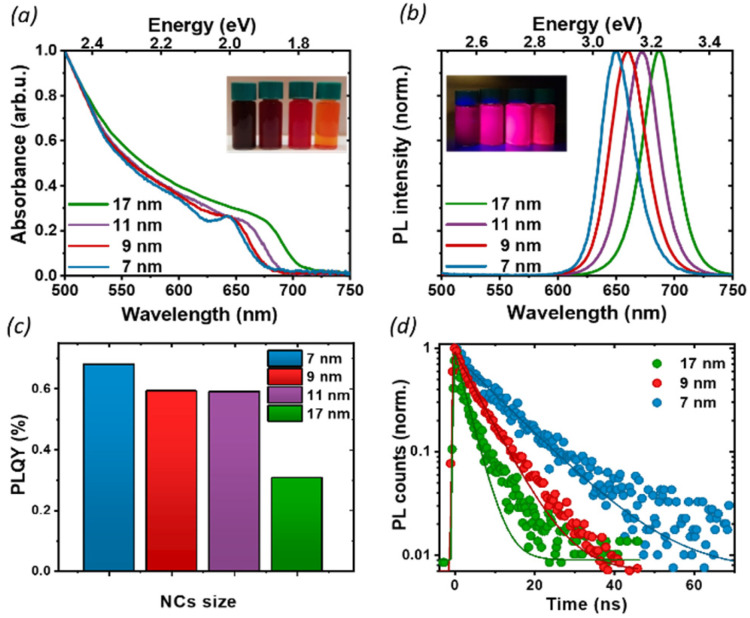
(a) Absorption, (b) PL spectra, and (c) absolute PLQY of nanocrystals of varying size, in toluene solution. We see a sharp band edge absorption in the red spectral region (a) and a PL spectrum (b) with a small Stokes shift and a narrow full width at half maximum (FWHM) around 35 nm. The PLQY was measured at 1.7 W cm^−2^. (d) TRPL decays for thin films of NCs of 3 different sizes at a fluence of 0.5 W cm^−2^. With an increase in the nanoparticle size, the PL lifetime is shortened.

We proceeded by investigating the optical properties of the four chosen solutions, looking for signatures of an excitonic population in their absorption and photoluminescence (PL) spectra (Fig. 2a and b). Due to the strong confinement inside the nanoparticles, optical excitation creates a stable exciton population with a large binding energy at room temperature, resulting in a sharp band edge absorption in the red spectral region (Fig. 2a) and a PL spectrum (Fig. 2b) with a small Stokes shift and a narrow full width at half maximum (FWHM) of 35 nm. Both PL and absorption spectra strongly depend on the particle size: as the NCs size decreases, the degree of quantum confinement experienced by the photo-excitation is increased, and as such the energy gap of the excitonic states is shifted to higher energies, resulting in a blue shift of absorption and PL emission. The particle size not only affects the luminescence color but also the absolute PL quantum yield (PLQY) (Fig. 2c): for an in-depth investigation, we considered the smallest and largest NPs (7 nm and 17 nm respectively) as extreme cases and selected the 9 nm NCs as an intermediate case study. The absolute PLQY was measured under CW excitation with a 405 nm laser, at a fluence of 1.7 W cm^−2^: the solutions showed a remarkable absolute quantum of yield up to 70% for the 7 nm NCs. As the NP volume increased, the PLQY was reduced from 70% to less than 40% at 17 nm. For the 9 and 11 nm NCs we measured very similar PLQY values(58% and 59% respectively); this can be explained in light of the relatively broad polydispersity compared to the small difference in their average size (see Fig. 1e), which does not allow one to identify different PLQY characteristics. Therefore, we decided to take the 9 nm NCs as a representative case for the intermediate size for the following in-depth analysis.

To better understand the correlation between the PLQY and the crystal size we performed time-resolved photoluminescence (tr-PL) measurements on drop-casted NCs films: the results are shown in Fig. 2d, where the PL decay has been fit with a single exponential decay. Particularly in larger particles, a small, long-lived component could be observed on top of the fast decay, with a decay time of the order of tens of ns. However, since it accounts for less than 5% of the observed amplitude decay, we are not considering its effects for the following discussion. An increase in the PL lifetime from 3 ns to 11 ns was observed as the nanocrystal size decreased. Together with the corresponding increase in the PLQY, this indicates a more significant contribution of the non-radiative recombination channel in the larger particles. To get an indication about the relative contribution of radiative and non-radiative recombination channels in different NPs we considered that the observed PLQY can be expressed as a ratio between the radiative recombination rate *k*_rad_, and total recombination rate *k*_tot_ = *k*_rad_ + *k*_non rad_. Hence from the measurement of the absolute PLQY = *k*_rad_/(*k*_rad_ + *k*_non rad_) together with the PL decay time *τ* = 1/*k*_tot_, we can estimate the radiative and non-radiative contribution to recombination rates (Table 1). This simple model does not aim at an exact quantitative determination, for which a much more detailed modeling of the recombination channels is required,^30^ but nevertheless, it allows for a comparison of the relative importance of radiative and non-radiative recombination channels across crystal sizes.

**Table 1:** Effect of the nanoparticle size on the relative importance of radiative and nonradiative recombination

NP size	*τ* (ns)	PLQY	*k*_rad_ (1/ns)	*k*_non-rad_ (1/ns)
7 nm	11.4 ± 0.1	0.68	60	30
9 nm	5.97 ± 0.05	0.59	100	70
17 nm	2.98 ± 0.05	0.31	110	240

Indeed, we found that while the radiative recombination rate is relatively similar going from 7 nm to 17 nm NCs, changing by less than a factor of 2, the non-radiative contribution is strongly size dependent, with an order of magnitude difference between the smallest and largest particles. This observation can be rationalized by considering the larger surface-to-bulk ratio in smaller crystals, where the binding to ligands ensures the passivation of the surface defect, thus reducing non-radiative losses and increasing the quantum yield. While for NCs with long ligands, aggregation does not represent a significant limiting factor, as shown also by TEM (Fig. 1), the comparatively smaller emissivity in larger NCs may stem from a less well-passivated surface as well as from a larger contribution from bulk defects.

**Fig. 3:**
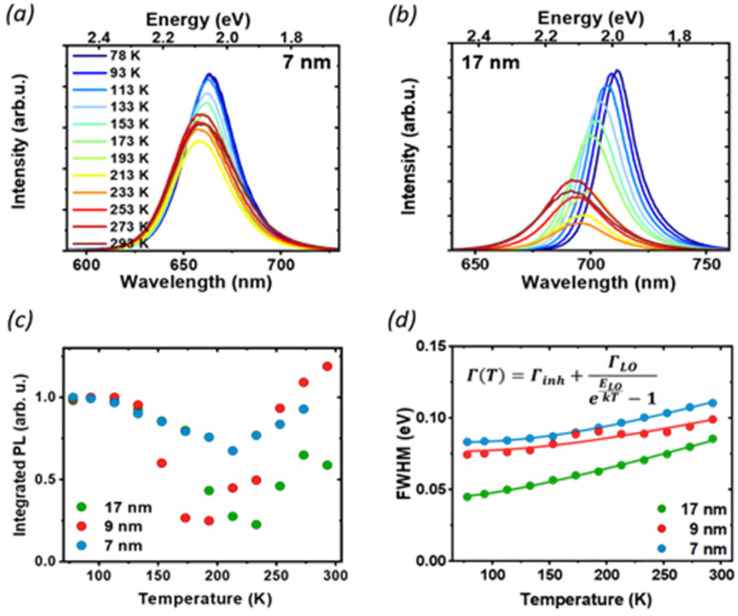
Temperature-dependent photoluminescence: PL spectra of 7 nm (a) and 17 nm (b) NCs as a function of temperature, under CW excitation at 405 nm. (c) Integrated PL intensity, normalized to the value at 77 K: the decrease in luminescence and subsequent recovery as the temperature is increased suggests the presence of non-radiative states that can be accessed at higher temperatures. (d) Temperature dependence of the PL FWHM and the corresponding fit with eqn (1).

To further understand the luminescence properties of CsPbI_3_ NCs, we exploited the temperature and pressure as thermodynamic variables to controllably manipulate the photophysical and structural properties of the nanoparticles. We focused first on temperature-dependent steady-state PL on drop-casted NC films. The PL measurements were conducted under vacuum, between 77 K and room temperature (Fig. 3 and Fig. S2†), a temperature range where the ligands we used are stable and any PL changes are fully reversible:^31^ with increasing temperature we observed a blue shift of the band-gap, broadening of the PL and a complex trend for the PL intensity. The difference in the temperature-dependent spectral change for the extreme cases of 7 nm and 17 nm (Fig. 3a and b) is striking: while the strongly confined 7 nm particle show only a moderate peak shift and relatively small changes in FWHM and intensity, the 17 nm NCs show a larger blue shift and broadening, together with a more pronounced change in the emission intensity. The PL peak energy *E*_g_(*T*) (Fig. S3†) is blue-shifted as the temperature increases across all particle sizes because of lattice thermal expansion, while the PL intensity shows a more complex thermal evolution with marked differences for different sizes. The integrated area under the PL spectrum is shown in Fig. 3c, normalized to its value at 77 K for the ease of comparison. From this value, the luminescence at first decreases with heating as scattering with a larger population of thermal phonons becomes more prominent, but then shows a recovery of intensity at temperatures higher than 200 K. Such effect is the most prominent for the 17 nm NCs, which show a significant temperature dependent quenching with 70% luminescence loss, as well as a smaller recovery compared to the 9 nm NPs. Conversely, the PL of the smallest NC is not as strongly affected, losing only 20% of the PL intensity upon heating. Temperature-dependent absorption spectra do not show similar drastic changes at 200 K (Fig. S4†), suggesting that there is no phase transition involved in the process, and rather points to the conclusion that the PL recovery is more likely due to the de-trapping from non-emissive states.

At low temperatures, defects capture carriers resulting in non-radiative recombination. As the temperature increases, the trapped species can gain enough energy from collisions with the surrounding environment to overcome the energy barrier for de-trapping and contribute to the emission, leading to the observed increase of PL intensity. The emerging picture is consistent with the conclusions drawn from static PL measurements: in larger particles, the contribution from bulk defects is more significant due to the smaller surface-to-volume ratio. This results in a decrease in the PLQY as well as the PL lifetime, and more significant changes in temperature-dependent quenching, as de-trapping becomes more prominent. However, significant changes in the intensity and linewidth with temperature also suggest a large involvement of electron–phonon coupling, whose strength can be studied *via* its effect on the temperature-dependent PL linewidth (Fig. 3d). The total PL linewidth arises from intrinsic inhomogeneities, acoustic phonon scattering, and optical phonon scattering.^32^ The main source of thermal variation for the PL width is the change in the phonon population. While the contribution from the acoustic phonon population is only significant at much lower temperatures, for *T* > 100 K the scattering with longitudinal optical (LO) phonons represents the dominant broadening mechanism:^33^ in our analysis we thus neglect contributions from acoustic phonons and consider only intrinsic inhomogeneities and exciton–longitudinal optical phonon coupling. These different mechanisms of scattering between charge carriers and phonons or impurities can be expressed by Segall's expression (eqn (1)):^34^

1 *Γ*(*T*) = *Γ*_inh_ + *Γ*_LO_/(*e*^*E*_LO_/*kT*^ − 1),

where *Γ*_inh_ is the inhomogeneous broadening constant, *Γ*_LO_ is the exciton–longitudinal optical phonon coupling coefficient, and *E*_LO_ is the energy of LO phonons. The fits (Fig. 3c, solid lines) are in good agreement with the measured data; the estimated model parameters are presented in Table 2.

**Table 2:** Temperature dependent FWHM: estimation of parameters related to exciton–phonon coupling

NPs size	*Γ*_inh_ (meV)	*Γ*_LO_ (meV)	*E*_LO_ (meV)
7 nm	82.8 ± 0.3	129 ± 14	43 ± 2.4
9 nm	76.0 ± 1.4	90 ± 11	41 ± 4.8
17 nm	43.7 ± 0.9	71 ± 12	26 ± 3.0

We found longitudinal optical phonon energies of 10s of meV, well in line with typical LO modes. The largest NCs have *E*_LO_ = 26 meV, in agreement with the values reported in the literature for phonon modes in CsPbI_3_;^25,35^ smaller particles, on the other hand, show larger phonon energies of around 40 meV. Such behavior can be explained in strongly confined systems, where the modes of comparable size with the NC can experience energy shifts due to the influence of surface modes.^35,36^ The energy of optical phonons is a significant parameter for assessing the effect of phonon scattering on radiative excitonic recombination: as the estimated exciton binding energy of CsPbI_3_ nanocrystals is 25 meV for sizes close to 17 nm,^37^ phonon scattering does not represent a significant exciton dissociation path at cryogenic temperatures, but might be detrimental under room temperature conditions, particularly for larger nanocrystals.^25^

Inhomogeneous broadening has a strength between 44 and 83 meV, while broadening due to phonon coupling is the dominant contribution, with values from 71 to 129 meV. Both the inhomogeneous broadening and LO-phonon coupling are reduced in larger particles. For inhomogeneous broadening, this can be related to a more significant size variability of smaller NCs and to the larger contribution from the surface, where ligand distribution might be uneven. The temperature-dependent PL broadening is instead mostly due to EP interaction: here again, we found a more pronounced effect of phonon coupling in particles smaller than 10 nm, which is also corroborated by the trend in exciton–phonon coupling retrieved from the fitting of the PL peak shift (see Fig. S3 and the parameter *A*_EP_ in Table S1†). In NCs phonon propagation can be disrupted by scattering from the nanocrystal surface, causing phonon confinement within the nanograin^38^ and enhancing the coupling of exciton with lattice phonons in smaller particles.^39^ In addition, bonding with ligands has also been shown to enhance EP coupling:^40^ again, a larger contribution of the bulk can explain the lack of the observed enhancement for less strongly confined NCs.

**Fig. 4:**
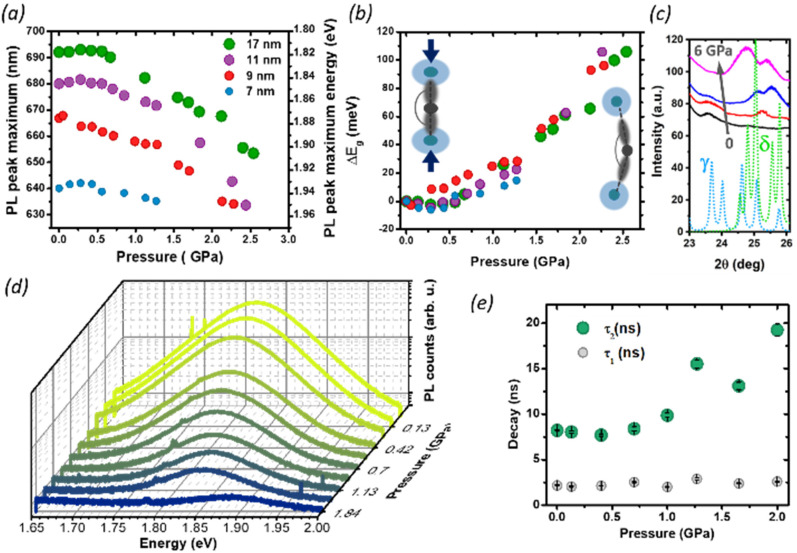
PL pressure response of CsPbI_3_ NCs of different sizes: (a) the PL peak shift of CsPbI_3_ NCs with sizes of 7, 9, 11, 17 nm. (b) Shift of the PL energy with pressure. Insets: schematic representation of the deformation mechanisms of Pb–I bonds. When the pressure initially increases, the Pb–I bonds start to shorten, which increases the orbital overlap and results in band-gap narrowing and red shift of the PL. Beyond 0.35 GPa, a further increase in the pressure starts to tilt the octahedra, decreasing the Pb–I–Pb angle (<180°) and decreasing the overlap of the electronic wave functions of Pb and I, causing an increase in the band-gap and the PL blue shift. (c) Pressure dependent XRD. The continuous lines show the experimental data at 0 GPa (black), 0.13 GPa (red), 1.85 GPa (blue), 6.14 GPa (magenta). The dotted lines show the calculated diffraction pattern for the CsPbI_3_ γ-phase (light blue) and the δ-phase (green). With increasing pressure, the peak at 23.7° (belonging to the γ-phase) gradually disappears, while two new peaks at 25.1° and 25.6° appear indicating the formation a new formed δ-phase. (d) Representative pressure-dependent PL emission spectra of 11 nm NCs, showing gradual quenching of the PL intensity with pressure. (e) Time-resolved photoluminescence results: evolution of PL kinetics as a function of pressure, for 9 nm NCs. Exponential data are fitted with bi-exponential eqn (2) extracting fast and slow decay time.

We then studied the size-dependent optical response of the NCs to the application of hydrostatic pressure by placing drop-casted nanocrystals in a diamond anvil cell (DAC). Given the soft nature of the perovskite lattice, the application of pressure is an ideal post-synthesis method to investigate the structure–property relationship by adjusting interatomic distances, lattice deformation, and band-to-band electronic overlap.^15,18,41–44^ For all nanocrystals in this study, the PL peak energy initially remained nearly constant up to 0.35 GPa (Fig. 4a), with a minimal red-shift of about 1–5 meV (Fig. 4b), followed by an abrupt blue shift at a higher pressure up to 2.5 GPa. Considering the specific composition of the metal halide perovskite band structure, such change can be ascribed to two different deformation modes of the crystal lattice.^15,18,21,29^ For CsPbI_3_, the valence band maximum (VBM) originates from the antibonding interaction of Pb 6s and I 5p electronic orbitals while the conduction band minimum (CBM) arises primarily from the I 5p orbitals and Pb 6p orbitals.^45^ The reduction of the unit cell volume at higher pressures proceeds in the first phase with the compression of the lead iodide octahedra: the reduced Pb–I bond length enhances the orbital overlap bringing the Pb 6s and I 5p orbitals closer, destabilizing the VBM and pushing it to higher energies.^18^

The CBM, on the other hand, is largely unaffected by octahedral compression because of the nonbonding character of the CBM, and small changes in the Pb–I distance will not have a destabilizing effect: the resulting band gap narrowing rationalizes the initial red-shift seen at low pressures. Similar considerations apply to the PL redshift observed at lower temperatures, which follows the lattice contraction (Fig. 3 and Fig. S3†). Conversely, above 0.35 GPa, the lattice deformation through the tilt of PbI_6_ octahedra becomes dominant, and the lower Pb–I–Pb bond angles decrease the electronic band dispersion opening the band-gap with a consequent blue-shift of the PL.^9,10,42^ Previous reports have shown that small CsPbI_3_ NCs possess a lower octahedral tilt, being closer to a cubic symmetry than their bigger counterparts,^13^ and this might affect their deformation behavior under pressure. While the trend in the PL peak shift is retained for all NCs sizes, we note that the rate of change of the peak position with the pressure above 0.35 GPa is smaller for the smallest NCs (7 nm). A more drastic change, however, occurs in the PL quenching behavior. During the whole pressurization cycle, the PL intensity is gradually quenched before finally disappearing (Fig. 4c), and the pressure-induced change is fully reversible in the investigated range of pressures (Fig. S5†). While darkening occured at around 2.5 GPa for the biggest size NCs, we observed the total PL quenching of 7 nm NCs already at 1.25 GPa. To understand the origin of this behavior, we performed pressure dependent XRD analysis focusing on 17 nm NCs, which provided the best diffraction intensity (Fig. S1†). In the range 24°–27° we probed an evolution of the diffraction peaks with the gradual appearance of a new pattern at high pressures, suggesting the occurrence of a solid–solid phase transition from the initial orthorhombic perovskite (γ phase) to the δ phase. While there are a few reports suggesting the loss of long-range order at high pressures,^10,18,41,46^ in our case we did not find clear signs of amorphization in our measurement range up to 6 GPa. The results are in good agreement with the changes we observed in the PL intensity. The δ phase, known as the yellow phase, is a poorly emissive non-perovskite structure with a much larger bandgap of about 3.0 eV; while at mild pressures there is still enough emissive γ phase to obtain a strong PL signal, at a higher pressure the non-emissive δ phase takes over and the luminescence rapidly diminishes until quenching. As we expect the phase transition to proceed from a nucleation site in the NCs,^7,47^ the fluctuation length scale triggering the structural change could be shorter in smaller NCs, where the higher surface-to-volume ratio could also allow greater surface fluctuations of the octahedral tilt favoring structural rearrangements.^8^ This supports why PL quenching occurs much earlier for 7 nm NCs than for bigger sizes. Finally, we investigated the effect of structural deformations on the exciton recombination through time-resolved PL measurements as a function of pressure, focusing on the intermediate NC size (9 nm, Fig. S6 and S7†). As the pressure is increased, the PL at first decays more rapidly, but at pressures higher than 1 GPa the luminescence clearly becomes more long-lived and cannot be well described by a single exponential decay. To accommodate data at all pressures, the PL decays were fit with a double exponential equation

2 *I*(*t*) = *A*_1_*e*^−*t*/*τ*_1_^ + *A*_2_*e*^−*t*/*τ*_2_^

where *A*_1_ and *A*_2_ are the corresponding fractional amplitudes, *τ*_1_ and *τ*_2_ are the fast and slow decay times, respectively. The results are reported in Table S2:† the fast decays occur typically in a couple of ns and are relatively independent of pressure, while the slow decay times vary from 7 to 20 ns. Interestingly, the evolution of the PL kinetics (Fig. 4e) follows closely the trend of the PL peak shift (Fig. 4d), and can be equally divided into two main trends: in the Pb–I compression region (<0.35 GPa) the lifetime becomes shorter with the increase in the pressure, while a marked prolongation of the lifetime is observed at higher pressurization. This is not consistent with the defect formation upon compression, which leads to a decrease of the carrier lifetime,^48,49^ possibly indicating structural robustness of the nanoparticles against defect formation. The increase in the lifetime is instead consistent with the lattice distortion occurring at a higher pressure: as the Pb–I–Pb bond angles are deformed, so is the band structure, resulting in a loss of overlap between the electronic states responsible for exciton formation. This results in a reduction of the recombination rate^50–52^ and consequently an increase in the PL lifetime, stressing the key role of controlling the structural properties to modulate the recombination dynamics of these complex semiconductors.

## Conclusions

In this work, we have investigated the size-dependent photophysical properties of CsPbI_3_ nanocrystals (in the range of 7–17 nm) by employing temperature and pressure as thermodynamic variables to controllably modulate the energetic and structural parameters of the system. Our observations of the temperature-dependent PL peak energy and linewidth highlight the influence of both defects and electron–phonon interaction on efficient luminescence, and their relation with NC sizes. This observation stresses the need for a careful consideration of the chosen ligands, as we find that the level of surface coverage not only directly affects the availability of non-radiative loss channels but also affects the luminescence of the NPs through more subtle phonon coupling effects, which increase in strength in smaller nanoparticles. Since electron–phonon coupling sets a fundamental intrinsic limit to charge transport, it is intimately related to the optoelectronic properties and thus to technological applications;^53^ understanding its dependence on the NC size and how to control it provides useful synthetic guidelines for tailoring NCs for light-emitting applications. By studying the pressure response, we have identified fundamental structure-properties relationships of the NCs: we observed a solid–solid transition from the γ-phase to the δ-phase at high pressures with a transition pressure that becomes lower for smaller size NCs, and likely contributes to the progressive PL quenching at high pressures, and further demonstrate that the lattice deformation mechanism strongly affects the bandgap and recombination dynamics of the material stressing the importance of the structural engineering of this class of soft semiconductors.

## Materials and methods

### Materials

All materials were used as received unless specified. 1-Octadecene (technical grade 90%), methyl acetate (anhydrous, 99.5%), oleylamine (technical grade 70%), silicone oil (viscosity 100 cSt), and cesium carbonate (99%) were purchased from Sigma-Aldrich. Lead(ii) iodide (99.99%, trace metals basis) was purchased from TCI. Oleic acid (technical grade 90%) was purchased from Alfa Aesar, *n*-hexane (for HPLC-Isocratic grade-Reag.Ph.Eur) from CARLO ERBA Reagents S.A.S.

### Solution preparation and thin film deposition

The precursor solution (Cs-oleate) was swiftly injected into a hot solution of 1-octadecene (ODE) and PbI_2_ salt, carboxylic acids (oleic acid (OA)) and primary amine (oleylamine (OAm)). When the PbI_2_ precursor dissolved completely, N_2_ flow was introduced into the flask heating it up to the desired temperature (140 °C–220 °C). Then the Cs-oleate precursor was swiftly injected into the reaction flask. Within just a few seconds from injection, the solution became turbid red. Further crystal growth and nucleation were stopped by immersing the reaction flask into an ice bath. After cooling to room temperature, the size-selective precipitation and purification of CsPbI_3_ NCs were introduced using Methylammonium acid (MeOAc). The precipitate was collected and re-dispersed in *n*-hexane. Solutions were filtered and then kept in a refrigerated vial. CsPbI_3_ NCs were first deposited as thin films prepared by spin coating the colloidal solution onto a glass substrate and drying it under N_2_ flow multiple times, to ensure adequate substrate coverage. The successful thin film deposition was validated by the comparison of PL spectra of the films with those of the NCs in solution (Fig. S8†).

### Solution characterization

Details of TEM measurements, and the characterization of the static absorption and luminescence and absolute luminescence quantum yield of the solutions are given in the ESI.†

### Nanocrystal characterization

The luminescence properties of NC thin films were characterized under different temperature and pressure conditions using a Linkam cryostat and a Diamond Anvil Cell (One20DAC). Further details of the steady state and time-resolved PL measurements are given in the ESI.† The effect of pressure on the NC structure was also investigated by XRD.

## Author contributions

O. V. and G. F. contributed equally to this work. O. V. synthesized the nanocrystals and contributed to the spectroscopic and structural characterization. G. F. performed the time-resolved photophysical characterization and supported the data analysis. E. L. W. performed the luminescence quantum yield experiments. L. L. acquired the TEM images. G. T. performed the pressure-dependent XRD experiments. M. D. A. worked on the data analysis. A. P. and D. C. supervised and coordinated the work. O. V., G. F., D. C., and A. P. conceived the experimental idea. All authors contributed to the final version of the manuscript.

## Conflicts of interest

There are no conflicts to declare.

## Supplementary Material

NR-015-D2NR06345J-s001
