# Supplementary material for: Structural effects on the luminescence properties of CsPbI3 nanocrystals
Source: Nanoscale. 2023 Feb 14;15(12):5712–9. doi: 10.1039/d2nr06345j (PMC10035506; doi:10.1039/d2nr06345j)
Supplement: NR-015-D2NR06345J-s001 [file NR-015-D2NR06345J-s001.pdf]

Electronic Supplementary Material (ESI) for Nanoscale.
This journal is © The Royal Society of Chemistry 2023

Structural effects on the luminescence properties of $CsPbI_3$ nanocrystals

Olivera Vukovic [1,2,3], Giulia Folpini [1], E Laine Wong [1], Luca Leoncino [4], Giancarlo Terraneo [5], Munirah D. Albaqami[6], Annamaria Petrozza [1,6]* Daniele Cortecchia [1,#,*]

[1] Centre for Nano Science and Technology (CNST@PoliMi), Istituto Italiano di Tecnologia, Via Pascoli 70, Milan 20133, Italy

[2] Molecular Materials and Nanosystems & Institute for Complex Molecular Systems, Eindhoven University of Technology, 5600 MB Eindhoven, The Netherlands

3 Université de Pau & Pays Adour, CNRS, IPREM UMR 5254, 2 Avenue du Président Angot, Pau F-64053, France

[4] Electron Microscopy Facility, Istituto Italiano di Tecnologia, Via Morego 30, Genova 16163, Italy

[5] Laboratory of Supramolecular and Bio-Nanomaterials (SupraBioNanoLab), Department of Chemistry, Materials, and Chemical Engineering "Giulio Natta", Politecnico di Milano, via L. Mancinelli 7, 20131 Milano, Italy

[6]Chemistry Department, College of Science, King Saud University, Riyadh 11451, Saudi Arabia

[#] Present Address: Dipartimento di Chimica Industriale "Toso Montanari", Università di Bologna, 40136 Bologna, Italy

*annamaria.petrozza@iit.ti; daniele.cortecchia2@unibo.it

Materials and methods.

Synthesis and sample preparation

Cs_2CO_3 precursor: In a 25 ml three-neck flask, first we added 0.200 g of Cs_2CO_3 (cesium carbonate), 10 ml of ODE (1-octadecene) and 0.65 ml of OA (oleic acid). This was degassed at 120°C for 30 min under vacuum to form Cs-oleate precursor solution. After 30 min N_2 flow was introduced into the flask and solution was kept under N_2 until was completely dissolved in the solution.

PbI_2 precursor: The PbI_2 precursor was formed by mixing 0.250 g of PbI_2 (lead iodide) and 12.5 ml of ODE in a three-neck flask and heated at 120°C for 30 min under vacuum. While the solution was still under vacuum, 1.25 ml of preheated OA and 1.25 ml of preheated OA were quickly injected into the PbI_2 solution. OA and Oleylamine (OAm) helped with dissolving PbI_2 quickly, making previously turbid orange solution into transparent yellow.

Growth of $CsPbI_3$ nanocrystals. When the PbI_2 precursor dissolved completely, N_2 flow was introduced into the flask heating it up to the desired temperature (140˚C – 220 ˚C). Then 1 ml of the Cs-oleate precursor was swiftly injected into the reaction flask. Within just few seconds from the injection, solution became turbid red. Further crystal growth and nucleation was stopped by immersing reaction flask in an ice bath.

After cooling to room temperature, $CsPbI_3$ NCs size-selective precipitation and purification was introduced. Methyl acetate (MeOAc) was added in the initial $CsPbI_3$ NCs solution in a volume ratio of 3 (NCs):1 (MeOAc). After centrifuging at 7500 rpm for 5 min, larger NCs were precipitated. Precipitate was collected and redispersed into 3 ml of n-hexane and supernatant was decanted for the next step (MeOAc) was added again in the same ratio). This second centrifugation produced another batch of NCs with a smaller size, compering to the first one. This same process was repeated until there was no obvious precipitate of NCs from the supernatant. Average number of batches from one synthesis was three. Some of the final solutions required one more centrifugation (without adding extra MeOac) in order to discard supernatant which sometimes contained some bigger agglomerates or precursor crude leftovers. All solutions were was filtered through a PVDF/L0.22 µm filter and then kept in a vial inside the fridge (5˚ C). These solutions were stable for a month. For longer stability of the solutions, few months, they were kept in freezer (- 21˚C).

Thin film deposition. $CsPbI_3$ NCs were first deposited as thin films prepared by spin coating the colloidal solution onto a glass substrates and drying it under N_2 flow multiple times, to ensure adequate substrate coverage. The spectra of the films at room temperature are nearly identical with the PL spectra of the NCs in solution, validating the success of the deposition procedure (Fig. S8). Only deviation has been seen for the smallest, 7nm, NCs.

Transmission electron microscopy (TEM)

Transmission electron microscopy (TEM) for size distribution analysis was performed using a JEM-1400Plus (JEOL) equipped with a thermionic source (LaB6) and operated at 120 kV. Images were acquired by the means of Bright Field-TEM technique. The samples contained colloidal suspensions of $CsPbI_3$ NCs (size ranging from 7-17 nm) which were used in the further experiments reported here. Specimens were prepared by drop casting 5µl of additionally diluted solution in n-hexane (1:10 vol.), on the ultra-thin carbon film with Cu grid. Samples were dried 5-10 minutes at room temperature.

A statistics on NCs dimension was performed measuring the cube edge length of approximately 200 particles for each sample. The values were acquired manually with the help of Digital Micrograph software and collected in the histogram reported in Fig 1e. NCs average sizes match the expected values for 7 nm, 9 nm and 11 nm and 17 nm samples.

XRD

Spin-coated films of NCs on Si wafers were characterized by X-ray diffraction (XRD) using a BRUKER D8 ADVANCE with Bragg-Brentano geometry, Cu Kα radiation (λ = 1.54056 Å), step increment of 0.02° and 2 s of acquisition time. Pressure dependent XRD analysis on the 17 nm NCs were carried out Bruker APEX-II diffractometer equipped with sealed-tube and CCD detector, using Mo-Kα radiation (λ=0.71073 Å). NC samples were measured at room temperature. Specifically, the 17 nm NCs were load, in silicon oil, on Diamond Anvil Cell (DAC) with diamond anvil type Ia and culet of 0.4 mm. The internal pressure was measured using the ruby fluorescence method [1]. The NCs-loaded DAC was mounted on a standard goniometer head and centered to X-ray beam. The diffraction patterns at different pressures were collected on the CCD detector and the obtained two-dimensional ring patterns were integrated and converted to one dimensional powder profile using CrysAlis Pro program. Tested pressure: 0 GPa, 0.13 GPa, 1.85 GPa, 6.14 GPa.

Steady - state absorption and photoluminescence

Steady- state absorbance of perovskite solutions, in a quartz cuvette, was measured using a UV-VIS spectrophotometer Shimadzu uv-2700. Photoluminescence of the same solutions was characterized using a NanoLog Fluorometer (Horiba Jobin-Yvon), with excitation wavelength 450 mn and a 2 nm spectral resolution and using an iHR320 detector in the visible range.

Absolute PLQY

Absolute values of PLQY were obtained from measurements performed in an integrating sphere (Labsphere). Excitation was provided by a 405 nm c.w. diode laser and spectra acquired through an optical fiber coupled from the sphere to a spectrometer (Ocean Optics Maya Pro 2000) with an intensity of 10 mW. PLQY values were calculated employing the method proposed by deMello et al. [3]

Temperature dependent steady state absorption and photoluminescence

All temperature dependent measurements were performed under vacuum using a Linkam Stage cooled with liquid nitrogen. Steady state absorption spectra was measured on perovskite thin

films deposited on quartz using a UV/VIS/NIR spectrophotometer Lambda 1050, Perkin Elmer. Photoluminescence was excited using a 405 nm Oxxius laser focused on the sample with a 10 cm lens. PL was detected using a Maya1000 visible spectrometer.

Pressure- dependent measurements

All pressure-dependent measurements were performed on a Diamond Anvil Cell (One20DAC) with diamond anvil type Ia and culet of 0.4 mm. Stainless still gasket, with the pre- indented hole, was centered on one of the diamonds. Sample was loaded by drop casting several times and drying under a nitrogen flux between the depositions. When the sample was dried, pressure marker (ruby powder) and pressure transmitting medium (silicon oil) were placed into the hole in the gasket. Silicon oil was applied as a pressure- transmitting medium in order to establish hydrostatic conditions. The pressure was measured by using the ruby fluorescence method.[1] With applying pressure ruby photoluminescence peaks are shifting toward higher wavelengths (Fig. S9) and by fitting peaks with Lorentzian profiles it is possible to determinate the exact pressure.[2]

The high- pressure evolution of steady-state photoluminescence spectra was collected using a micro Raman confocal microscope (via Raman Microscope Renishaw, 50 x objective, 532 nm excitation wavelength).

Time-resolved PL (tr - PL)

TRPL in the ps range was performed using a Hamamatsu streak camera and a Coherent Chameleon oscillator (pulse duration 30 fs, repetition rate 80 MHz) as a pump, using a pump wavelength λ = 400 nm obtained by frequency doubling the fundamental at 800 nm in a BBO crystal. An acousto-optic modulator (AOT) was used to reduce the laser repetition rate to 2 MHz. The measurements were performed using a measurement window of 50 ns for pressures up to 1 GPa, and a 100 ns widow for higher pressure (respectively 0.5 ps and 1 ns temporal resolution). The sample was pressurized in a DAC, on the previously described way, and light was focused on NCs clusters through a 50x long working distance microscope objective, (r = 2 μm), corresponding to a typical of pump fluence of 150 mW/cm^2.

Calculation of radiative and non-radiative decay rates

The radiative and non-radiative decay rates, respectively k_{rad} and k_{nonrad}, are calculated by combining tr-PL results with absolute PLQY. First we note that the total decay rate $k_{tot} = k_{nr} + k_{rad} = 1/\tau$ is the inverse of the effective decay time τ, which for this simple model can be

retrieved from fitting the tr-PL decay with a single exponential. Furthermore, the absolute PLQY is the ratio of radiative recombination processes with all decay paths, namely PLQY = k_{rad} / k_{tot}. Hence, the radiative decay rate can be retrieved from the experimental data as k_{rad} = PLQY* k_{tot} = PLQY/τ Subsequently, the non radiative decay rate can be found as k_{nonrad} = k_{tot} - k_{rad} = (1-PLQY)/τ.

Table S1. Parameters obtained from the temperature-dependent PL peak fitting

NC size	$E_0(T = 0)$ eV	A_{TE} 10^{-4} eV K^{-1}	A_{EP} eV
**7 nm**	1.856	1.97 ± 0.78	-0.066 ± 0.041
**9 nm**	1.809	2.29 ± 0.63	-0.067 ± 0.026
**17 nm**	1.721	5.01 ± 0.54	-0.050 ± 0.010

Table S2. Parameters obtained from the double-exponential fit of the pressure dependent PL decays.

Pressure	A_1	τ_1 (ns)	A_2	τ_2 (ns)
**0 GPa**	0.35 ± 0.02	2.3 ± 0.1	0.65 ± 0.02	7.9 ± 0.2
**0.13 GPa**	0.65 ± 0.02	2.28 ± 0.08	0.4 ± 0.01	8.2 ± 0.2
**0.4 GPa**	0.75 ± 0.01	2.07 ± 0.04	0.25 ± 0.01	7.0 ± 0.1
**0.7 GPa**	0.84 ± 0.02	2.52 ± 0.06	0.25 ± 0.02	7.9 ± 0.25
**1 GPa**	0.75 ± 0.02	1.99 ± 0.06	0.25 ± 0.02	6.3 ± 0.2
**1.27 GPa**	0.75 ± 0.02	2.8 ± 0.1	0.25 ± 0.02	12.1 ± 0.5
**1.65 GPa**	0.8 ± 0.04	2.0 ± 0.2	0.2 ± 0.03	8.5 ± 0.6
**2 GPa**	0.81 ± 0.03	3.3 ± 0.2	0.19 ± 0.03	16.4 ± 1.8

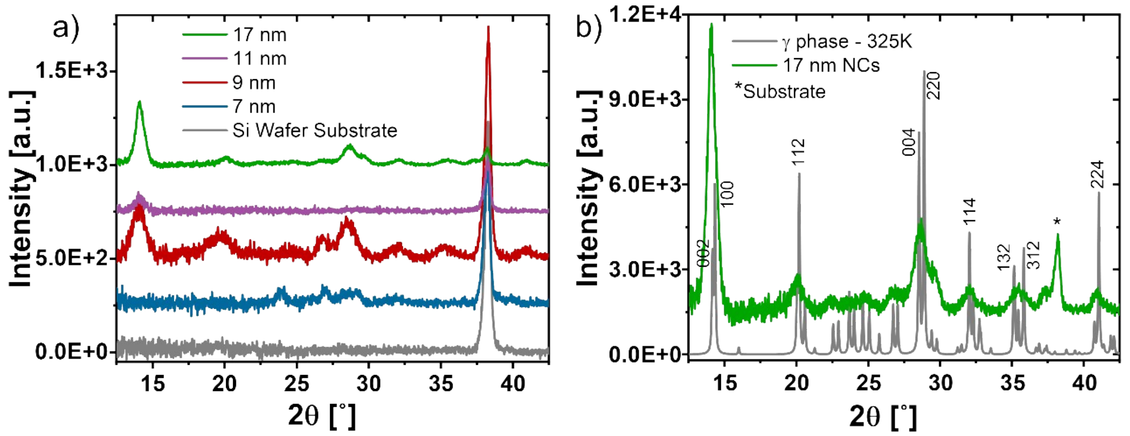

Figure S1. a) X-ray powder diffraction (XRD) pattern of $CsPbI_3$ NCs of four different solutions (7, 9, 11 and 17 nm) spin coated on a Si wafer substrate. Differences of scattering intensities between different samples are due to differences in the amount of material that was possible to collect and deposit on the Si substrate following the size-selective precipitation. b) Comparison of the XRD experimental pattern of the 17 nm NCs with the simulated XRD pattern for the $CsPbI_3$ γ phase (325 K), showing an excellent match in good agreement with the most recent reports. [4] The main diffraction peaks are indexed on the figure.

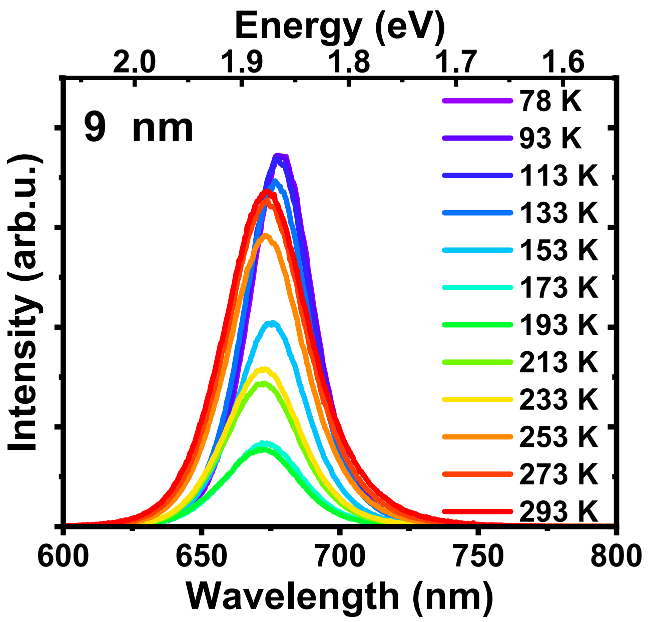

Figure S2. Temperature dependent PL spectra, for the 9 nm NCs (7 and 17 nm are found in Fig.3a,b): the graph indicates three main phenomena with decreasing temperature: anomalous

blue band gap shift (but characteristic for perovskite), narrowing of the PL and a variation in PL intensity, with inflection points are observed at around 200 K.

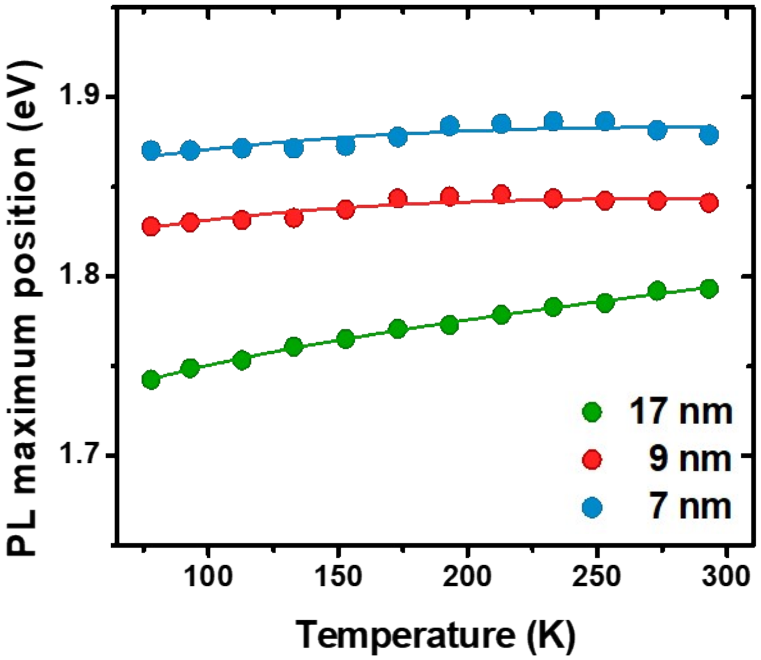

Figure S3. Change of PL peak position with temperature. The PL peak energy $E_g(T)$ is blue shifted as the temperature increases across all particle sizes.

The relative shift is related to electron–phonon (EP) interaction and lattice thermal expansion through a single oscillator model $E_g(T) = E_0 + A_T T + A_P\left[2\Big/\left(e^{\frac{\hbar\omega}{kT}} - 1\right) + 1\right]$, [5] where E_0 is the un-renormalized bandgap, A_{TE} and A_{EP} are the weight of the thermal expansion and carrier-phonon interaction, respectively, and $\hbar\omega$ represents the average optical phonon energy. Results are reported in Table S1. As expected, due to quantum confinement the normalized band gap $E_g(T=0) = E_0 + A_{EP}$ decreases with NC size. The thermal expansion A_{TE} on the other hand is increased. The value of the average optical phonon energy was fixed to the values obtained in the fitting of the temperature dependence of the PL FWHM (see Fig. 3 and Table 2 of the main text).

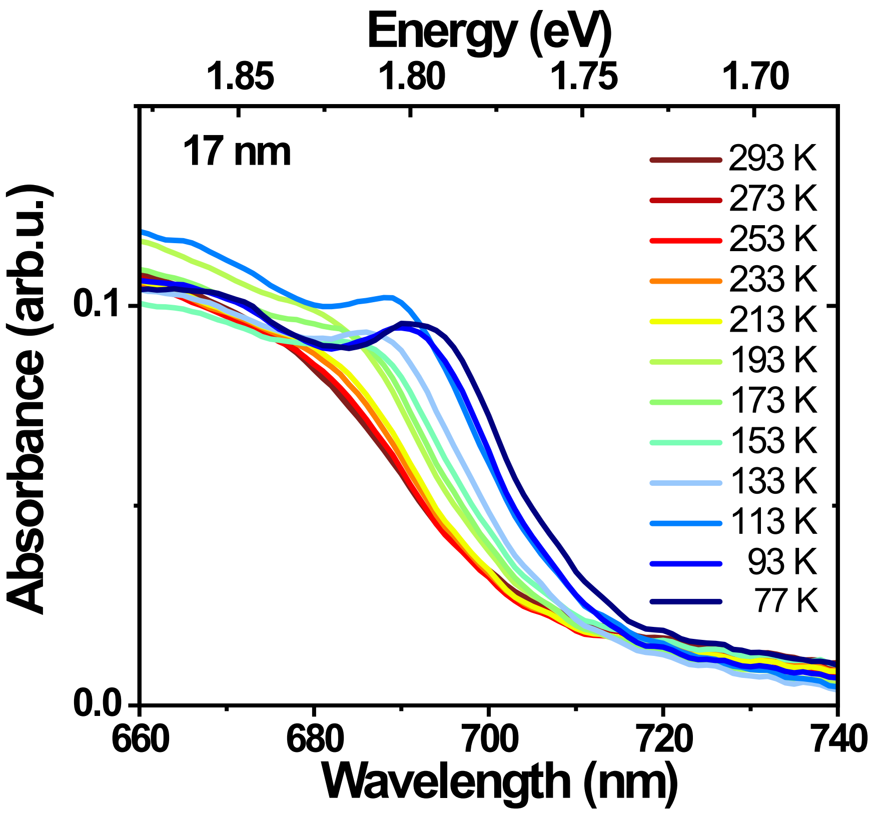

Figure S4. Temperature dependent absorbance of the 17 nm NPs: no phase transition is observed between 77 K and 300 K.

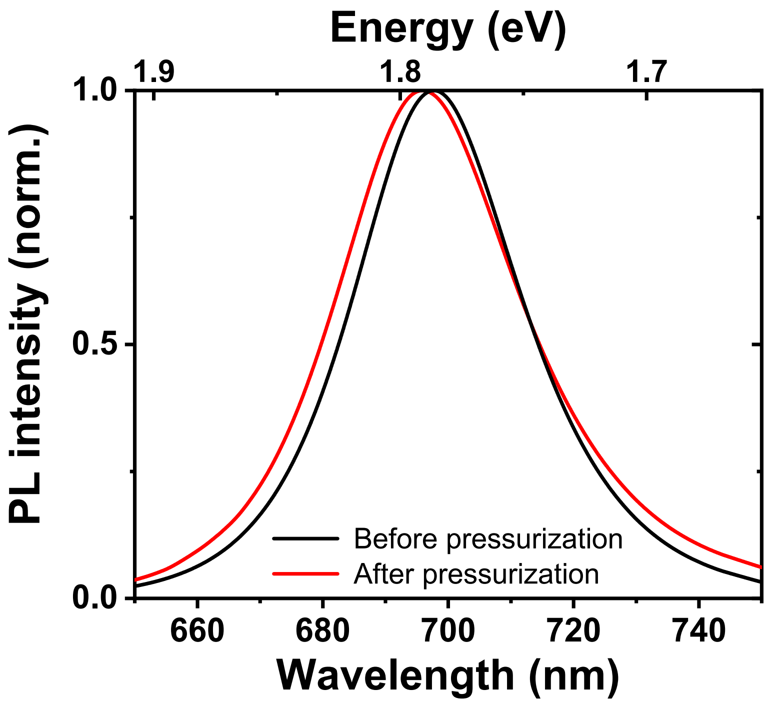

Figure S5. PL spectra recorded for 17 nm NCs at atmospheric, before a pressurization cycle (black line) and after release of the highest applied pressure (red line). During the whole pressurization cycle, the PL intensity is gradually quenched before finally disappearing, but the pressure-induced change is fully reversible in the investigated range of pressures.

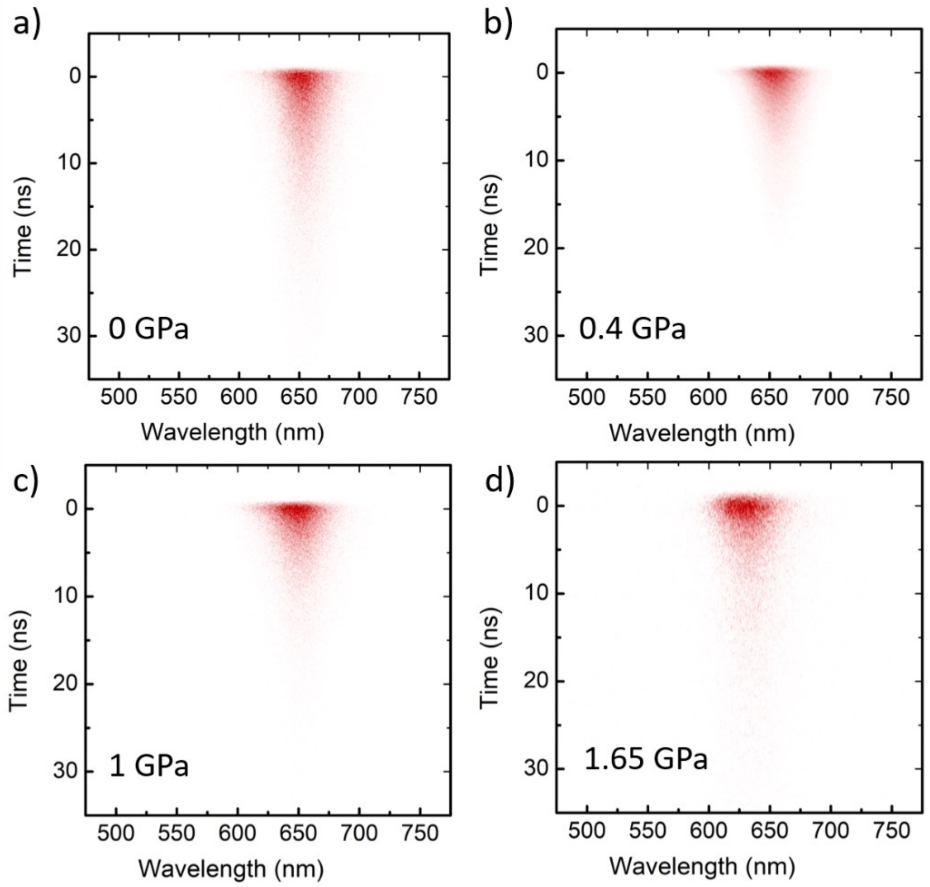

Figure S6. Representative TR-PL data at different pressures, showing the luminescence maps as detected in time-resolved single photon counting mode.

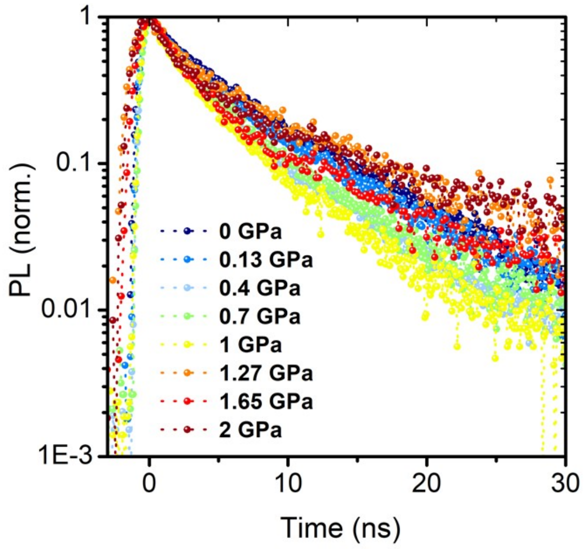

Figure S7. Evolution of PL kinetics as a function of pressure for the intermediate size particles (9 nm): the initial decrease in lifetime upon pressurization is then reversed at even higher

pressures. The data is retrieved by the TR-PL maps by integrating the PL spectra in the 600 –

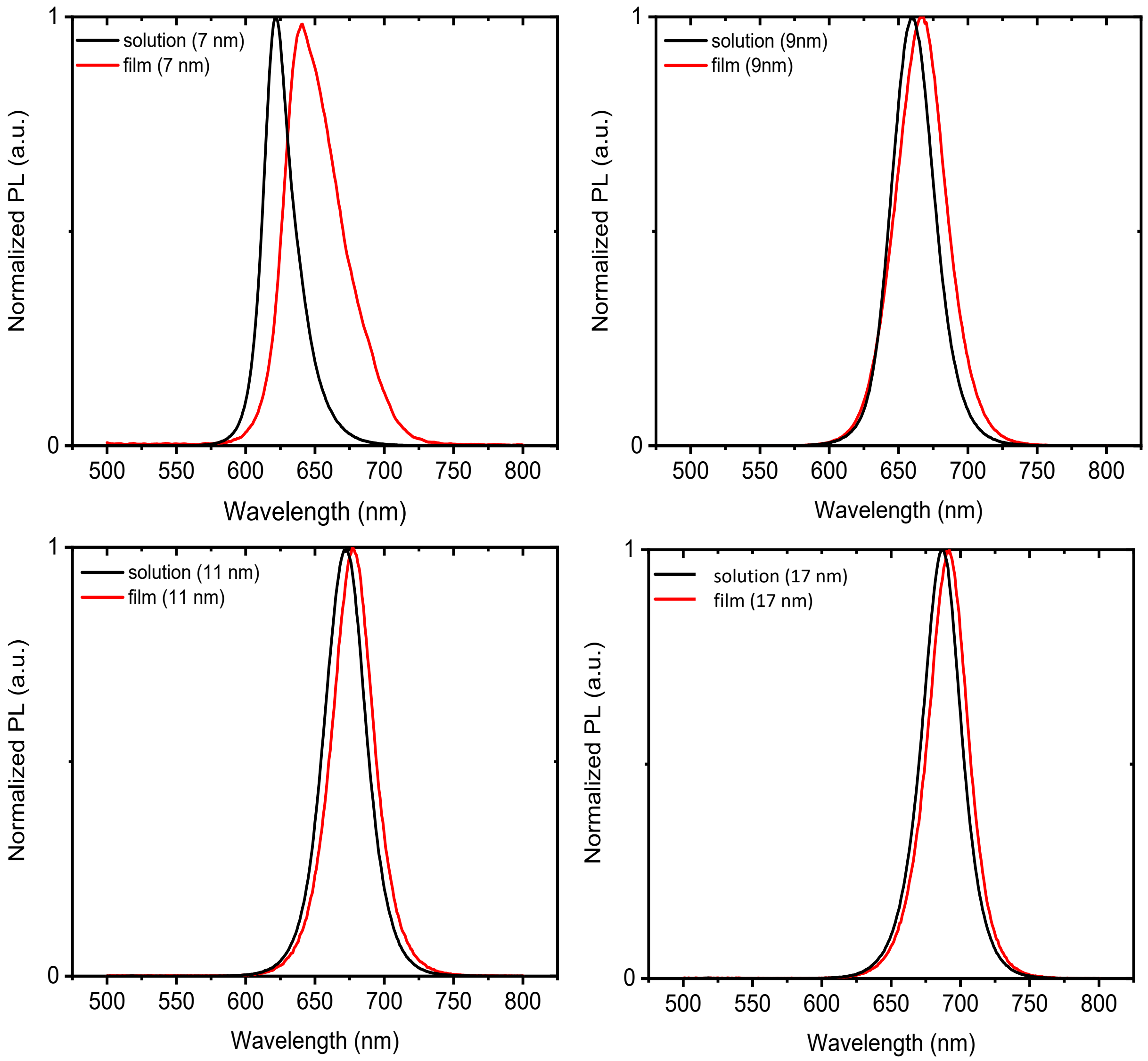

675 nm region.

Figure S8. PL spectra recorded for different sizes solutions (black lines) and films spin coated from these solutions (red line). The spectra of the films at room temperature are nearly identical with the PL spectra of the NCs in solution, validating the success of the deposition procedure. Only deviation has been seen for the smallest, 7 nm, NCs.

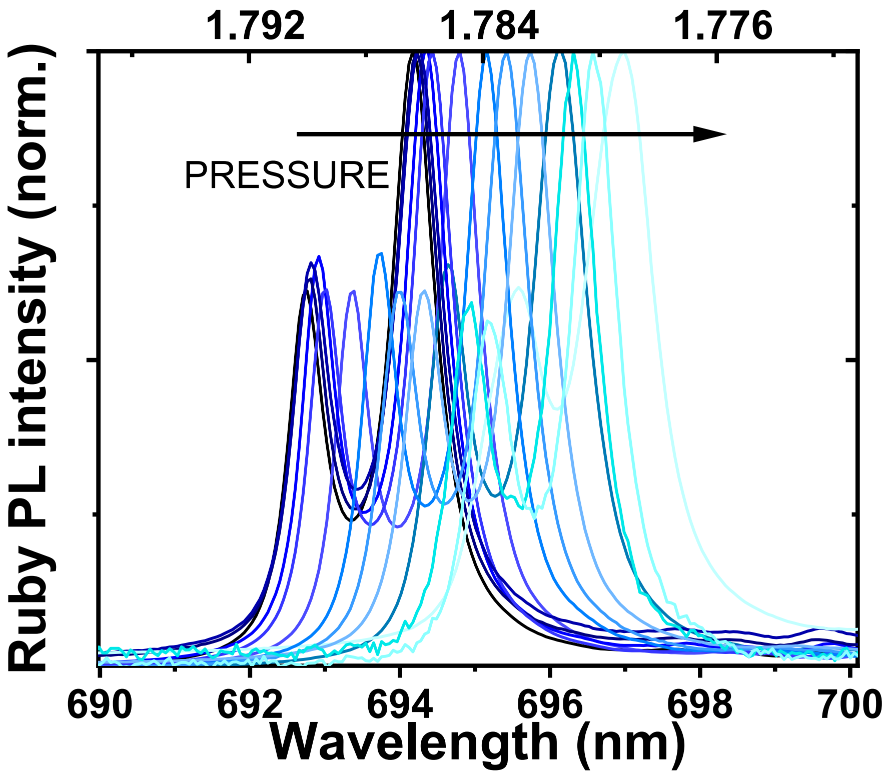

Figure S9. PL spectra recorded for ruby powder under the differen pressures. With applying pressure ruby photoluminescence peaks are shifting toward higher wavelengths and by fitting peaks with Lorentzian profiles it is possible to determinate the exact pressure.

References

[1] H. K. Mao, J. Xu, and P. M. Bell, "Calibration of the ruby pressure gauge to 800 kbar under quasi-hydrostatic conditions," *J. Geophys. Res.*, vol. 91, no. B5, p. 4673, 1986.

[2] K. Syassen, "Ruby under pressure," *High Press. Res.*, vol. 28, no. 2, pp. 75–126, 2008.

[3] and R. H. F. de Mello, John C., H. Felix Wittmann, "An Improved Experimental Determination of External Photoluminescence Quantum Efficiency."

[4] J. C. B. Ii, L. M. G. Hall, P. Tongying, G. Dukovic, and J. M. Weber, "Pressure Response of Photoluminescence in Cesium Lead Iodide Perovskite Nanocrystals," 2018.

[5] K. E. W. Ei, Z. X. U. Hongjie, R. U. C. Hen, X. I. N. Z. Heng, and X. I. C. Heng, "Temperature-dependent excitonic photoluminescence excited by two-photon absorption in perovskite CsPbBr 3 quantum dots," vol. 41, no. 16, pp. 16–19, 2016.
